# Crystallizing Student-Interest in Biochemistry

**DOI:** 10.1063/4.0001073

**Published:** 2025-10-27

**Authors:** Selina S Huang, Susanna S Huang

**Affiliations:** 1 STARS research society, Marietta, Georgia, USA; 2 Georgia Institute of Technology, Atlanta, Georgia, USA

## Abstract

Crystallography is a highly important and integral field of science. Through crystallography, we can crystallize a multitude of substances from inorganic to organic to even macromolecular molecules. By crystallizing these macromolecular molecules, like DNA and proteins and using x-ray diffraction, we can determine their structure and in turn, reveal their functions. With the elucidation of their structures and functions, inhibitors can be designed to target macromolecules related to diseases, so that the adverse effects of diseases, such as cancers, can be reduced.

While crystallography can be used to perform cutting-edge research and discover treatments to diseases, it can also be used to induce scientific creativity and interest in the student community, especially in elementary and middle school students. The growth of large, faceted crystals is an intrinsically rewarding experience as the crystals are inherently beautiful and attractive. At STARS (Structural Nucleic Acid Anticancer Research Society), we hope to harness the aesthetic properties of crystals to spur the scientific passion and creativity of K-12^th^ grade students. Through crystal growing workshops, competitions, and summer camps, we share our enjoyment and love for crystallography with younger students. These amazing experiences allow them to see in-person the beauty and excitement of crystallography.

In these experiences, we prepare crystal-growing instructional videos and lessons for students, introduce how to grow glow-in-the-dark UV crystals, teach how to record detailed crystal growth observations, and explain how to make a seed crystal, among many other skills. Moreover, these skills encompass many of the same fundamental skills that any scientist would need for being successful in research. Learning these observational and note-taking skills, following the scientific method, utilizing trial and error, encouraging collaboration, testing precision and accuracy, and spurring patience and confidence are all important fundamental skills to establish within these students. In the last four years, STARS has hosted a variety of crystallography activities for elementary and middle school students in the form of summer camps, competitions, and workshops. We have hosted three crystallography virtual summer camps during this time. Moreover, STARS, in collaboration with Timber Ridge Elementary School and Dodgen Middle school, hosted the 2021 Timber Ridge Crystal-Growing Competition, 2022 Cobb County Crystal- Growing Competition, and the 2023 Dodgen Crystal-Growing Competition. The elementary students were very excited about growing crystals and had so much fun growing colorful salt and sugar crystals. Meanwhile, the middle school students had so much fun learning about the purpose of crystallography, growing the creative crystals for the competition, and playing Kahoots with king-sized candy bars as prizes. With the generous sponsorship from the ACA, STARS hosted an award ceremony with numerous prizes and awards prepared for the student winners to celebrate their hard work and perseverance in their crystal-growing activities and endeavors. This year, we will be hosting a crystallography workshop for Dodgen Middle School students in May where we introduce to younger students not only how to grow inorganic crystals but also how to micropipette. We hope that with our continued efforts in hosting these local crystal-growing activities and competitions, we may further spread our love and passion for crystallography to more young students and future scientists in our community, sharing with them the significance of crystallography in a fun yet exciting way.

